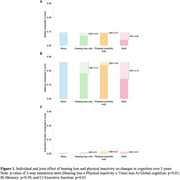# Physical inactivity and hearing loss – a dangerous duo for dementia

**DOI:** 10.1002/alz70860_105255

**Published:** 2025-12-23

**Authors:** Surim Son, Mark R. Speechley, Guangyong Zou, Manuel Montero‐Odasso

**Affiliations:** ^1^ Schulich School of Medicine & Dentistry, Western University, London, ON, Canada; ^2^ Schulich School of Medicine & Dentistry, Division of Geriatric Medicine, Western University, London, ON, Canada

## Abstract

**Background:**

Recent lifestyle intervention trials showed that it is possible to improve cognition by combating combinations of risk factors. Importantly, these risk factors cluster in individuals, and specific combinations of these risk factors can have synergistic effect. However, the effects of specific combinations and how they differ from other combinations have not been explored. Additionally, the interactions among risk factors are not well understood. We aimed to identify the combinations of modifiable risk factors that are highly prevalent and assess their detrimental effect on cognitive change, as well as their synergistic effect.

**Method:**

The prevalence of dyad combination of the 12 modifiable risk factors were estimated using the Canadian Longitudinal Study on Aging (*N* = 30,097). Of the five most prevalent combinations for each combination type, the pooled and synergistic effects of the identified combinations on 3‐year cognitive changes were examined using linear mixed models. The synergistic effect was examined by evaluating whether the joint effect of risk factor combinations over time is greater than the sum of individual effects, using an interaction term. All analyses were stratified by sex and 2 age groups – midlife (45 to 64) and later life (65‐85).

**Result:**

Most Canadians (95%) had at least one risk factor, with 80% having two or more, 58% having three or more, and 35% having four or more risk factors. The combination of hearing loss and physical inactivity was the most prevalent dyad with the largest effect size (mean difference = ‐0.07 SD; 95% CI: ‐0.09 to ‐0.06; *p* <0.001; d = ‐0.28). A significant synergistic interaction was observed between hearing loss and physical inactivity (*p* = 0.005). The observed joint effect of hearing loss and physical inactivity (mean difference = ‐0.08) was larger than the expected joint effect of hearing loss and physical inactivity on cognitive improvement (mean difference = ‐0.04; Figure 1).

**Conclusion:**

Identifying the most prevalent risk factor combinations with the greatest potential effect size can help strategically tailor multidomain dementia interventions for maximum impact. Dementia prevention and risk reduction programs should prioritize improving auditory health and increasing exercise levels.